# Tiliroside Ameliorates Ulcerative Colitis by Restoring the M1/M2 Macrophage Balance *via* the HIF-1α/glycolysis Pathway

**DOI:** 10.3389/fimmu.2021.649463

**Published:** 2021-03-31

**Authors:** Hongda Zhuang, Qi Lv, Chao Zhong, Yaru Cui, Luling He, Cheng Zhang, Jun Yu

**Affiliations:** ^1^ Translational Medicine Centre, Jiangxi University of Traditional Chinese Medicine, Nanchang, China; ^2^ Jiangsu Key Laboratory for Functional Substance of Chinese Medicine, Stake Key Laboratory Cultivation Base for TCM Quality and Efficacy, School of Pharmacy, Nanjing University of Chinese Medicine, Nanjing, China; ^3^ Center for Metabolic Disease Research and Department of Physiology, Lewis Katz School of Medicine, Temple University, Philadelphia, PA, United States

**Keywords:** HIF-1α, glycolysis (glycolytic pathway), macrophage polarization, ulcerative colitis, tiliroside

## Abstract

Macrophages polarized to different phenotypes critically contribute to colitis development by coordinating inflammatory and anti-inflammatory processes. Herein, targeting the balance between the pro-inflammatory M1 and the anti-inflammatory M2 macrophage phenotypes can be a novel therapeutic approach for colitis. In the present study, we firstly demonstrated that tiliroside possessed the ability to alleviate the clinical symptoms of colitis as evidenced by decreased disease activity index (DAI) scores, longer colon length, reduced myeloperoxidase (MPO) activity, and improvement of colonic pathological damage *in vivo*. Furthermore, we showed that tiliroside modulated the balance between M1 and M2 macrophages toward a more anti-inflammatory status in colonic lamina propria but has little effect on the T cell population and epithelial barrier function in colitis mice. The macrophage depletion study further showed the protective effect of tiliroside was macrophage dependent *in vivo*. Mechanistically, our study demonstrated that tiliroside regulated cellular metabolism by inhibiting aerobic glycolysis in LPS and IFNγ stimulated macrophages. At the molecular level, tiliroside facilitated the proteasomal degradation of HIF-1α and downregulated mRNA expressions of HIF-1α dependent glycolytic enzymes in macrophages. Collectively, our data highlight the aberrant M1/M2 macrophage polarization in the initiation and development of ulcerative colitis and put forth the stage for considering tiliroside as a metabolic regulator in reprogramming macrophage polarization, which may serve as a promising therapeutic approach for treatment of inflammation-associated and metabolic disorders.

## Introduction

Ulcerative colitis (UC) is the most common inflammatory bowel disease that has arisen since the improvement of hygiene and urbanization at beginning of the 20th century in North America and Europe. It has recently begun to spread to developing countries with middle–high income, such as China (1). Characterized by constant abdominal pain, diarrhea, and hematochezia, the inflammation of UC affects the innermost layer of the lining of the intestine ranging from the colon to the rectum (2). While medical management (including 5-aminosalicylic acid, corticosteroids, immunosuppressants, biological agents, and probiotics) have been used to mitigate the symptoms successfully, these treatments are limited by high relapse rate and inevitable side effects, such as nausea, vomiting, myelosuppression, probability of infection, and risk of carcinogenesis (3, 4). Therefore, the development of novel and effective UC therapies is urgently required.

Innate immune system-mediated inflammation has been implicated in the initiation and development of UC. In assessing evidence for optimizing UC management, the focus is generally on anti-inflammatory agents that attune the intestinal immune response (5). As the central coordinator of colonic innate immunity, macrophages can be polarized into either classically activated pro-inflammatory (M1) or alternatively activated (M2) anti-inflammatory macrophages depending on the stimulation (6). The M1 macrophages are induced by pathogen-associated molecular patterns (PAMPs) such as bacterial lipopolysaccharide (LPS) and Th1 cytokines such as Interferon γ (IFNγ), producing a wide range of cytokines, such as TNF-α, IL-1β, IL-6, and inducible nitric oxide synthase (iNOS), to aggravate inflammation. In contrast, the M2 macrophages are induced by Th2 cytokines such as IL-4 and IL-13, and they possess the ability to express arginase-1 (Arg1), chitinase-like 3 (also called Ym1), and IL-10 to promote reparative processes and relieve inflammation (7). Furthermore, Meaghan M. et al. have illustrated that patients with UC have abundant M1 macrophage in the inflamed colon, whereas M2 macrophages are significantly enriched in non-inflamed ileum (8). Therefore, restoring the balance between M1 and M2 macrophage is crucial to the treatment of UC and the maintenance of colonic homeostasis.

Accumulating evidence has revealed that alterations in cellular metabolism are critical for supporting the different paradigms and functions of macrophages. For example, in M1 macrophage, the glycolysis and the pentose phosphate pathways are substantially increased to support the functions of anti-infection and production of inflammatory mediators (9). Conversely, macrophages differentiated with IL-4 and IL-13 induce transcriptional programs associated with long-chain fatty acid oxidation and oxidative mitochondrial metabolism to maintain the functions of restoration and release of anti-inflammatory cytokines (10). Furthermore, genetic or pharmacological inhibition of these metabolic pathways attenuates the acquisition of effector responses. Viral knockdown of carnitine palmitoyl transferase-1 (cpt1), the key enzyme that participated in fatty acid oxidation, reduced expression of M2 macrophage-associated cell surface markers (11). Blocking the glycolysis pathway with 2-deoxy-D-glycose (2-DG) treatment significantly inhibited the differentiation of pro-inflammatory macrophages after stimulation with LPS and IFNγ (12). These studies have provided compelling evidence that cellular metabolism is integral to macrophage polarization and targeting these metabolic pathways may be a promising strategy for the regulation of macrophage polarization.

Tiliroside is a flavonoid contained in several edible plants or specific plant parts (fruits, leaves, or roots), and previous studies have revealed its anti-inflammatory *in vivo* and *in vitro* experiments (13). *In vitro*, tiliroside could inhibit the production of TNF‐α, iNOS, and IL-6 in LPS-stimulated RAW264.7 and BV2 microglia (14, 15). In addition, tiliroside suppressed the production of PGE2 and the activation of NF-κB and MAPK signaling pathways (16). *In vivo* research demonstrated that intragastric administration of tiliroside markedly attenuated the mouse paw edema induced by phospholipase A2 and the mouse ear inflammation induced by 12-*O*-tetradecanoylphorbol 13-acetate (17). Although several studies have proved its anti-inflammatory biological activity, the effects of tiliroside on UC and the underlying mechanisms are still unclear. In the present study, we showed the protective effect of tiliroside on dextran sodium sulfate (DSS)- and 2, 4, 6-trinitrobenzene sulfonic acid (TNBS)-induced colitis in mice and uncovered a novel mechanism of tiliroside-regulating macrophage polarization *via* HIF-1α-dependent reprogramming of the glucose metabolic pathway.

## Materials and Methods

### Animals

Female C57BL/6 mice and Male BALB/c mice (6**–**8 weeks old) were purchased from the Hunan SJA Laboratory Animal Co., Ltd. (Hunan, China). All mice were housed with free access to diet and water in plastic cages at room temperature (24**–**26 °C) and kept on a 12 h light/dark cycle in a specific pathogen-free environment. The animal experiments were strictly performed in accordance with the Guide for the Care and Use of Laboratory Animals. All experimental procedures were approved by the Animal Ethics Committee of Jiangxi University of Traditional Chinese Medicine.

### Induction and Treatment of UC

To establish the DSS-induced colitis model, C57BL/6 mice were treated with 2.5% DSS (M.W. = 36, 000**–**50, 000: MP Biomedical, Solon, OH, USA) for 7 days followed by 3 days of normal drinking water. To establish the TNBS-induced colitis model, BALB/c mice were lightly anesthetized with isoflurane, and 2 mg of TNBS (Sigma, Wicklow, Ireland) dissolved in 40% ethanol was slowly administered into the lumen of the colons by using the soft catheter. Then, the mice were positioned head-down for 1 min to distribute the solution over the colon.

**Figure 1:**
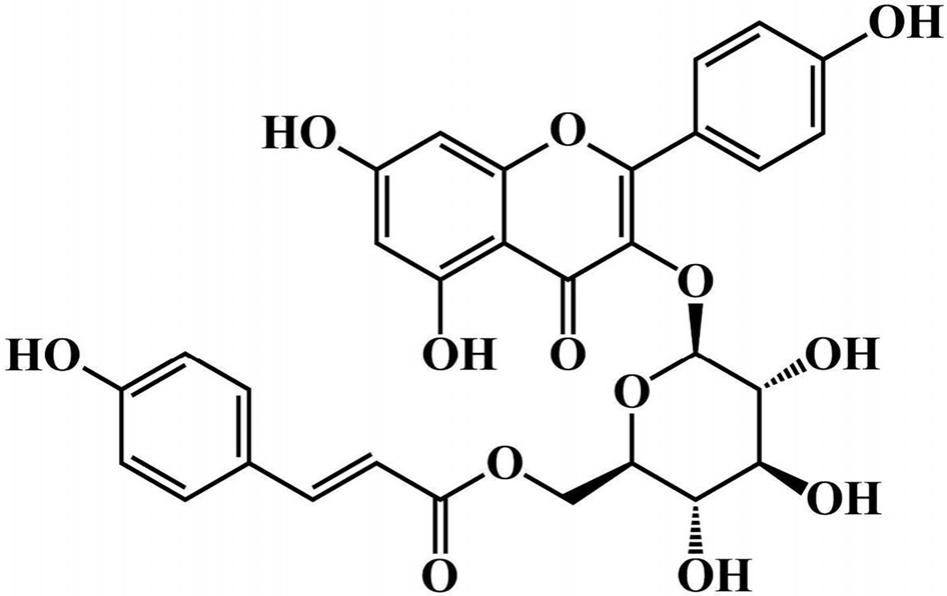
The chemical structure of tiliroside.

To evaluate the protective effect of tiliroside on colitis, the mice were randomly divided into the following six groups: Normal group, Model group, Tiliroside (12.5, 25.0, and 50.0 mg/kg) (**Figure 1**. purity > 98%: Chengdu Desite Bio Technology, Chengdu, China) groups, and 5-aminosalicylic acid (5-ASA, 200.0 mg/kg) (Ipsen Pharma, Shanghai, China) group. The tiliroside and 5-ASA were dissolved in 0.5% CMC-Na and orally administered by gastric gavage. Colon tissues were collected on day 10 and stored at -80°C until further analysis.

### Disease Activity Index (DAI)

The body weight, diarrhea, and rectal bleeding were measured every day, and the disease activity index (DAI) scores were calculated using the well-established system: (a) body weight loss: 0 points = none; 1 points = 1-5%; 2 points = 5-10%; 3 points = 10-15%; 4 points = over 15%; (b) stool consistency: 0 points = normal; 2 points = loose stools; 4 points = diarrhea; (c) gross bleeding: 0 points = normal; 2 points = hemoccult; 4 points = gross bleeding (18).

### Myeloperoxidase (MPO) Activity

The segments of the proximal colon (40 mg) were washed, minced, and homogenized with cold PBS. The activity of MPO in colons was measured using MPO activity kits (Nanjing JianCheng Bioengineering Institute, Nanjing, China) according to the manufacturer’s instructions.

### Histological Scores of Colon Tissues

The distal colon of each mouse was fixed in 4% paraformaldehyde, embedded in paraffin, sectioned and then stained with hematoxylin and eosin (H & E) (Beijing Solarbio Science & Technology, Beijing, China). The histological score was graded as follows (19): (a) inflammation severity (0 points = none, 1 points = mild, 2 points = moderate, 3 points = severe); (b): inflammation extent (0 points = none, 1 points = mucosa, 2 points = submucosa, 3 points = transmural); (c): crypt damage (0 points = none, 1 points = base 1/3 damage, 2 points = base 2/3 damage, 3 points = crypt lost but surface epithelium present, 4 points = crypt and surface epithelium lost); (d) proportion of tissue involvement (1 points = 0**–**25%, 2 points = 26-50%, 3 points = 51**–**75%, 4 points = 76**–**100%).

### Quantitative PCR Assay

Total RNA was isolated from colons or cells cultured *in vitro* by using TRIzol extraction reagent and then reverse-transcribed into cDNA by using HiScript Q RT SuperMix (Vazyme Biotech, Piscataway, NJ, USA). The qPCR was carried out using Hieff^®^ qPCR SYBR Green Master Mix reagent (Yeasen Biotech, Shanghai, China) according to the manufacturer’s instructions. The primers used were listed in **Supplementary Table 1**. The relative amount of each gene was normalized to the amount of β-actin and was then reported as the fold change of the basal level.

### Cell Culture

The human THP-1 cells were maintained in RPMI 1640 medium supplemented with 10% FBS, 4 mM glutamine, and 1 mM sodium pyruvate. For monocyte to macrophage differentiation, the THP-1 cells were seeded at a density of 3×10^5^ cells/mL and treated with the RPMI 1640 medium containing 100 ng/mL PMA for 2 days.

### Preparation of L929-Conditioned Medium

Mouse L929 fibroblasts were purchased from the Cell Bank of the Chinese Academy of Science (Shanghai, China). To obtain conditioned medium, they were cultured with an initial 90% confluence in RPMI 1640 medium (Beijing Solarbio Science & Technology, Beijing, China) containing 10% fetal bovine serum (FBS) (Gemini Biotech, California, USA) at 37°C and, 5% CO_2_ for 5 days. On day 6, the supernatants were harvested, filtered by using the strainer (pore size: 0.22 μM) and stored at -20°C for further experiment.

### Isolation and Culture of BMDMs

The bone marrow-derived macrophages (BMDMs) were isolated from femurs and tibias of C57BL/6 mice as previously described (20). Briefly, the bones were cut off at both ends and the marrow was eluted with RPMI 1640 medium by using sterile syringes. The elution was collected and centrifuged at 1600 rpm for 5 min. Subsequently, the supernatants were removed, and the cells were re-suspended in RMPI 1640 medium supplemented with 10% FBS under a humidified 5% (v/v) CO_2_ atmosphere at 37°C overnight.

The next day, the non-adherent cells, as well as loosely adherent cells, were harvested and centrifuged at 1600 rpm for 5 min. The supernatants were abandoned, and the cells were re-suspended with RPMI 1640 medium containing 10% FBS and 20% conditioned medium from mouse L929 fibroblasts. Cultured fluid was exchanged with fresh culture medium every 3 days. On day 7, the adherent cells were used for further studies.

### Macrophage Polarization

The BMDMs and PMA-differentiated THP-1 cells were treated with LPS (100 ng/ml) (Sigma, Wicklow, Ireland) and IFNγ (20 ng/ml) or IL-4 (20 ng/ml) (PeproTech, Rocky Hill, NJ, USA) to elicit M1 or M2 phenotype macrophages, respectively. After 24 h, cells were harvested and stained with fluorescein-conjugated anti-CD86 mAb or anti-CD206 mAb (Biolegend, London, UK). CD86-positive cells were obtained at over 90% in M1 phenotype macrophages (LPS & IFNγ stimulation). CD206-positive cells were obtained at over 95% in M2 phenotype macrophages (IL-4 stimulation).

### MTT Assay

The viability of BMDMs was evaluated by using Thiazolyl blue tetrazolium bromide (MTT) assay. Briefly, the BMDMs were seeded into 96-well plates and incubated with tiliroside (0, 10, 20, 40, and 80 µM) for 20 h. Then, 20 µL of MTT (5 mg/ml) (Beijing Solarbio Science & Technology, Beijing, China) was added into each well, and cells were continuously cultured for another 4 h. Lastly, the supernatants were removed, and 150 µL dimethyl sulfoxide (DMSO) was added into each well to dissolve the formazan crystals. The absorbance was determined at 570 nm by using Microplate Reader (Thermo, Waltham, MA, USA).

### Western Blotting

To prepare the whole protein lysate, BMDMs were lysed in NP-40 buffer supplemented with 1 mM PMSF on ice for 15 min. After centrifugation at 12000 rpm at 4°C for 10 min, the supernatant was carefully collected, and the protein concentration was determined by BCA Protein Assay Kits (Beijing Solarbio Science & Technology, Beijing, China) according to the manufacturer’s instructions. Subsequently, the samples were separated by 8% SDS-PAGE and transferred onto PVDF membranes (Millipore, Billerica, MA, USA). The membranes were blocked with 5% milk for 2 h at room temperature and incubated with different primary antibodies at 4°C overnight. The next day, membranes were incubated with the indicated secondary antibodies at room temperature for 1 h. Finally, the bands were visualized by using LI-COR Odyssey, and the signals were analyzed by using Image J software.

### Immunofluorescence Assay

The distal colon of each mouse was fixed in 4% paraformaldehyde, embedded in optimal cutting temperature, frozen in liquid nitrogen, and then sectioned. The 8μm-thick frozen section of the colon was firstly washed with PBS three times and then incubated with blocking buffer containing 1% bovine serum albumin (BSA), 10% goat serum, and 0.3% Triton X-100 at room temperate for an hour. Subsequently, the blocking buffer was removed, and the colon section was incubated with CD68 (Bio-Rad Laboratories, Cambridge, UK) and iNOS or CD206 antibody (Cell Signaling Technology, Inc. Danvers, MA, USA) overnight at 4°C. The next day, the specimens were rinsed with PBS three times and then incubated with corresponding fluorescent secondary antibody at room temperate for two hours. Finally, the specimens were counterstained with DAPI (Beijing Solarbio Science & Technology, Beijing, China) for 5 min at room temperate. Images were captured using a fluorescence microscope (Leica, Tokyo, Japan).

### Glycolytic Rate Assay

For glycolytic function assay, the BMDMs were plated on XFe24 cell culture microplates (Seahorse Bioscience, Santa Clara, CA, USA) for 7 days and then treated with LPS (100 ng/ml), IFNγ (20 ng/ml), and tiliroside (10, 20, and 40 μM) for 24 h. A total of 1 hour before the measurement, the cells were washed and the culture medium was replaced with Seahorse XF Base Medium supplemented with 10 mM glucose and sodium pyruvate (Seahorse Bioscience, Santa Clara, CA, USA). The extracellular acidification rate (ECAR) as parameters of glycolytic flux was measured on a Seahorse XFe24 bio-analyzer by using the XF Glycolytic Rate Test Kit (Seahorse Bioscience, Santa Clara, CA, USA) according to the manufacturer’s instructions. Briefly, cells were incubated in the Assay Medium and basal rates were recorded over three measurement periods. Next, Rot/AA (inhibitors of mitochondrial electron transport chain) were injected to inhibit mitochondrial oxygen consumption (and therefore CO_2_- derived protons). The second injection was 2-DG, which inhibits glycolysis through competitive binding of glucose hexokinase. The resulting decrease in proton efflux rate (PER) provides qualitative confirmation that the PER produced prior to the injection was primarily due to glycolysis. By using the Agilent Seahorse XF Glycolytic Rate Assay Report Generator, Convert ECAR measurements to PER, and mitochondrial OCR was used to determine the PER attributed to glycolysis (glycoPER) and mitochondrial (mitoPER) acidification.

### Glucose Uptake

Glucose uptake ability of BMDMs was evaluated by using the fluorescent glucose 2- [N-(7-nitrobenz-2-oxa-1, 3-diazol-4-yl) amino]-2-deoxy-D-glucose (2-NBDG) (Invitrogen Corp, Carlsbad, CA, USA). The BMDMs were seeded into 24-well plates at a density of 1 × 10^6^ cells/mL and then treated with LPS (100 ng/ml), IFNγ (20 ng/ml) and tiliroside (10, 20, 40 μM) for 24 h. After starvation for 2 h, cells were incubated with 2-NBDG (500 μM) for 1 hour. The cells were washed twice with PBS to remove any residual 2-NBDG before analyzing the amount of 2-NBDG uptake by using immunofluorescence assay.

### Lactate Content Measurement

The BMDMs were treated with LPS (100 ng/mL), IFNγ (20 ng/mL) and tiliroside (10, 20, 40 μM) for 24 h. Then, the lactate levels in the culture medium were determined by using a lactate assay kit (Nanjing JianCheng Bioengineering Institute, Nanjing, China) according to the manufacturer’s instructions.

### Statistical Analysis

Data were presented as the means ± S.E.M. Statistical analysis was performed by Prism 8 (GraphPad, CA, USA). Statistical differences were assessed by one-way or two-way analysis of variance test. A value of *P* < 0.05 is considered statistically significant.

## Results

### Tiliroside Treatment Attenuates Development of Colitis in Mice

**Figure 2:**
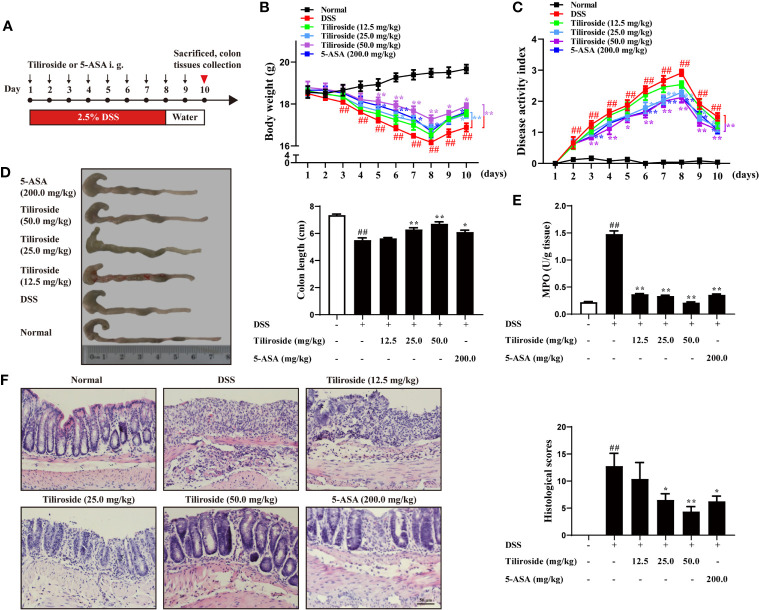
Tiliroside treatment attenuates DSS-induced colitis in mice. **(A)** Methods for DSS-induced acute colitis in C57BL/6 mice and tiliroside administration. **(B)** Body weight. **(C)** Disease activity index (DAI). **(D)** Colon length. **(E)** Myeloperoxidase (MPO) activity in colons. **(F)** Representative hematoxylin and eosin (H & E) staining of distal colon sections and corresponding histological scores (scale bar: 50 μm). Data were expressed as means ± S.E.M of eight mice in each group. *^##^P* < 0.01 *vs.* Normal group; ^∗^ *P* < 0.05 and ^∗∗^ *P* < 0.01 *vs.* DSS group.

To investigate the impact of tiliroside treatment on the development of colitis in mice, we firstly used a highly reproducible DSS-induced colitis mouse model that shares similar clinical signs with human UC and has been widely used as a preclinical model for drug development (21). C57BL/6 mice were fed with 2.5% DSS for 7 days followed by normal drinking water for another 3 days before tissue collection. Different dosages of tiliroside or 5-ASA were orally administered by gastric gavage from day 1 to 10 (**Figure 2A**). Compared with the DSS group, tiliroside (12.5, 25.0, and 50.0 mg/kg) and 5-ASA (200.0 mg/kg) significantly attenuated body weight loss, diarrhea, and rectal bleeding caused by DSS (**Figures 2B, C**). In addition, tiliroside (12.5, 25.0, and 50.0 mg/kg) and 5-ASA (200.0 mg/kg) also markedly prevented the shortening of colon length in colitis mice (**Figure 2D**). The MPO activity in colons was also significantly decreased upon tiliroside (12.5, 25.0, and 50.0 mg/kg) or 5-ASA (200.0 mg/kg) treatment (**Figure 2E**). Furthermore, histological analysis showed the tissue damage, including mucosal architecture, inflammatory cell infiltration, and crypt loss were markedly improved by tiliroside or 5-ASA treatment at the indicated dosage (**Figure 2F**). Given that the use of multiple models for pharmacodynamics validation is necessary for lead compound research, we then confirmed the protective effect of tiliroside on TNBS-induced colitis mice, another well-established colitis animal model. As shown in **Supplementary Figures S1A–D**, tiliroside (25.0 and 50.0 mg/kg) markedly alleviated the TNBS-induced colitis, evidenced by increased survival rate, longer colon length, decreased MPO activity, and ameliorated pathological changes of colons. Taken together, these results demonstrate that tiliroside possesses a potent preventive effect against colon inflammation.

### Tiliroside Modulates the Balance Between M1 and M2 Macrophage in Colitis Mice

**Figure 3:**
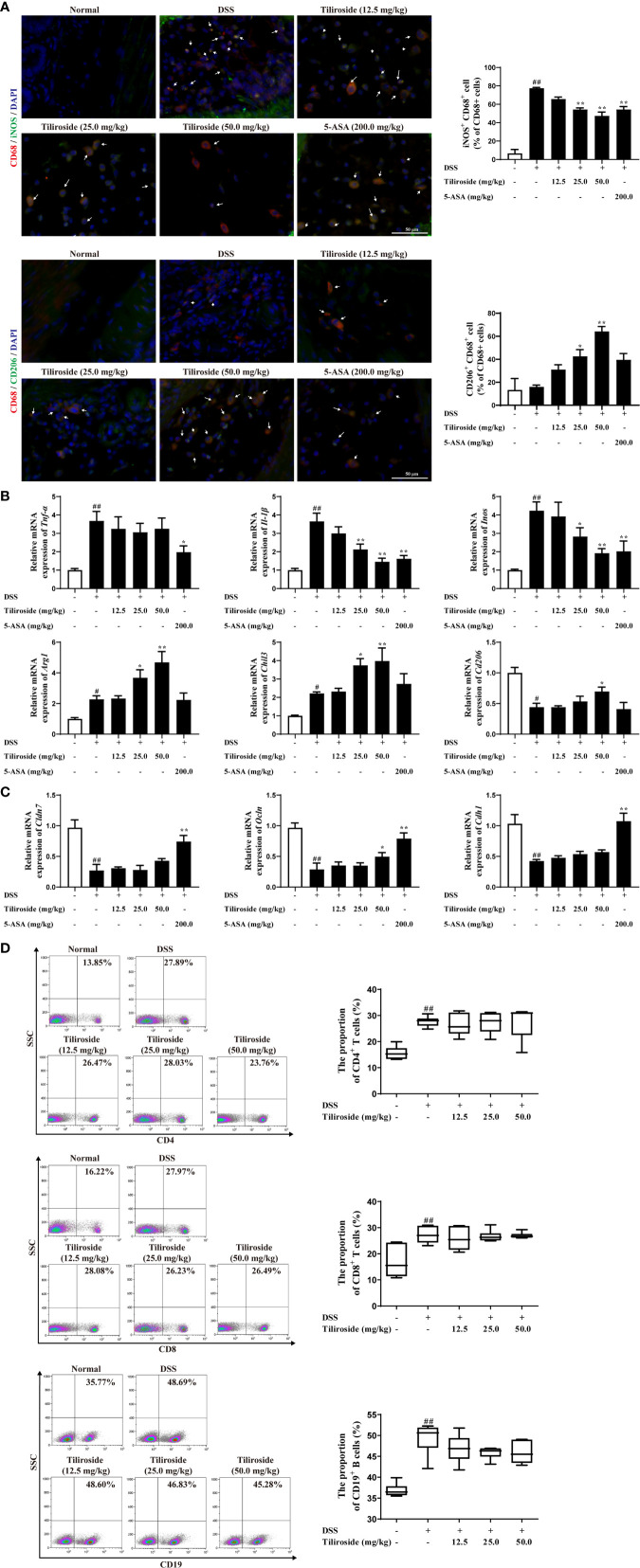
The effect of tiliroside on M1 and M2 macrophage balance *in vivo*. Mice were treated with 2.5% DSS for 7 days, followed by normal drinking water for another 3 days. The tiliroside (12.5, 25.0, and 50.0 mg/kg) or 5-ASA (200.0 mg/kg) were dissolved in 0.5% CMC-Na and then intragastrically administrated ​​for consecutive 10 days. **(A)** The expressions of CD68 (red), iNOS (green), and CD206 (green) in distal colon tissue were detected by using an immunofluorescence staining assay (scale bar: 50 μm). **(B)** The mRNA expressions of *Tnf-α*, *Il-1β*, *Inos*, *Arg1*, *Chil3*, and *Cd206* in colons were analyzed by using qPCR assay. **(C)** The mRNA expressions of tight junction proteins *Cldn7*, *Ocln* as well as the adherence junction protein *Cdh1* was detected by using the qPCR assay. **(D)** The proportion of CD8^+^, CD4^+^ T cells, and CD19^+^ B cells in lamina propria was detected by using flow cytometry. Data were expressed as means ± S.E.M of eight mice in each group. *^##^P* < 0.01 *vs.* Normal group; ^∗^ *P* < 0.05 and ^∗∗^ *P* < 0.01 *vs.* DSS group.

Emerging evidence supports the hypothesis that an excess of classic M1 macrophage and insufficient alternative M2 macrophage leads to severe colitis (22, 23). Therefore, we assessed whether tiliroside regulated the balance of M1 and M2 macrophage in colon tissues. As shown in **Figure 3A**, tiliroside (25.0 and 50.0 mg/kg) treatments significantly decreased the percentage of M1 macrophage phenotype (CD68^+^ iNOS^+^ double labeled) and upregulated M2 macrophage phenotype (CD68^+^ CD206^+^ double labeled) in the colonic lamina propria of colitis mice. Meanwhile, we analyzed the gene expression of M1/M2 specific factors in colons of colitis mice. The results showed that mRNA expressions of M1 macrophage-associated factors including *Il-1β*, and *Inos* were significantly decreased while the levels of M2 macrophage-associated markers including *Arg1*, *Chil3*, and *Cd206* were markedly increased with tiliroside (25.0, 50.0 mg/kg) treatments (**Figure 3B**). We then further investigated the effect of tiliroside on the epithelial barrier function and the proportion of CD4^+^ T cells, CD8^+^ T cell and CD19^+^ B cells, which have been demonstrated to play an important role in the occurrence and development of colitis (24–26). Tiliroside (12.5, 25.0, and 50.0 mg/kg) exerted little effect on the mRNA expressions of *Cdh1*, *Cldn7*, and marginally increased the expression of *Ocln* only at the dosage of 50.0 mg/kg (**Figure 3C**). Furthermore, the infiltration of CD4^+^ T cells, CD8^+^ T cells, and CD19^+^ B cells in the colonic lamina propria remained unchanged upon the tiliroside (12.5, 25.0, and 50.0 mg/kg) administration in the DSS-induced colitis mice (**Figure 3D**). Taken together, these data imply that tiliroside may alleviate colitis by selectively modulating the balance between M1 and M2 macrophages.

**Figure 4:**
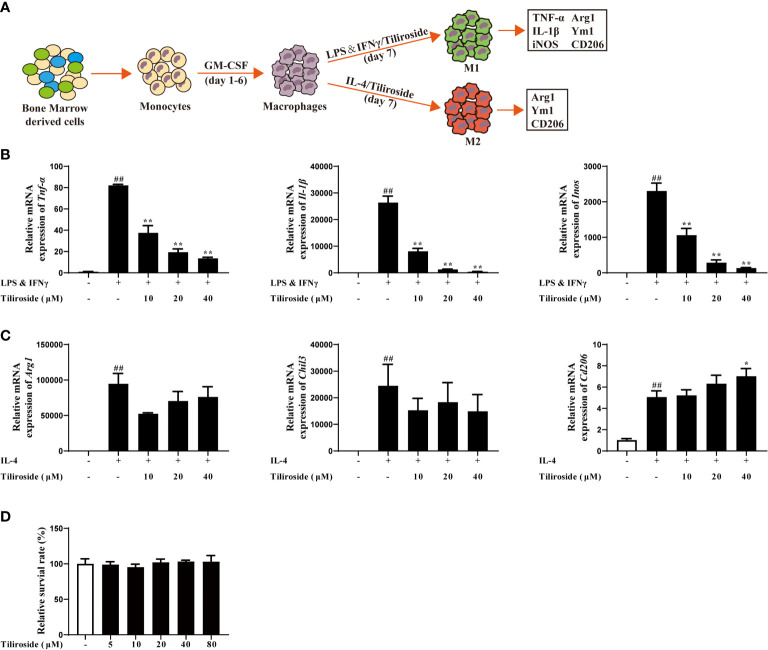
Tiliroside regulates macrophage M1 polarization. **(A)** Schematic of primary mice bone marrow monocyte to macrophage culture and subsequent polarization *in vitro*. **(B)** The BMDMs were treated with LPS (100 ng/ml) & IFNγ (20 ng/ml) and tiliroside (10, 20, and 40 μM) for 24 h, the mRNA expressions of *Tnf-α*, *Il-1β*, and *Inos* were analyzed by using qPCR assay. **(C)** The BMDMs were treated with IL-4 (20 ng/ml) and tiliroside (10, 20, and 40 μM) for 24 h, the mRNA expression of *Arg1*, *Chil3*, and *Cd206* were analyzed by using qPCR assay. **(D)** The viability of BMDMs was measured with MTT assay in response to different concentrations of tiliroside for 24 h. Data were expressed as means ± S.E.M of three independent assays *in vitro*. ^##^ *P* < 0.01 *vs.* Control group; ^∗^ *P* < 0.05 and ^∗∗^ *P* < 0.01 *vs.* LPS & IFNγ or IL-4 group.

Subsequently, we established the M1 and M2 macrophage polarization model *in vitro* to clarify how tiliroside regulates M1/M2 balance (**Figure 4A**). LPS (100 ng/mL) and IFNγ (20 ng/mL) induced mRNA expression of M1 marker genes, including *Tnf-α*, *Il-1β*, and *Inos*, were significantly attenuated by tiliroside (10, 20, and 40 μM) (**Figure 4B**). On the other hand, tiliroside had little effect on levels of IL-4 stimulated M2 marker genes *Arg1*, *Chil3*, and *Cd206* induction in BMDMs (**Figure 4C**). Tiliroside (10, 20, and 40 μM) had no effect on cell viability (**Figure 4D**). Similarly, the regulatory effects were observed in PMA-differentiated human THP-1 cells (**Supplementary Figure S2B**). A previous study has shown that GM-CSF could induce the formation of M1-like macrophage (27). We thus sought to investigate whether tiliroside has an effect on GM-CSF-induced M1 polarization or if it is LPS and IFNγ signaling dependent. Interestingly, tiliroside (10, 20, and 40 μM) exerted little effect on mRNA expressions of *Tnf-α*, *Il-1β*, and *Inos* in BMDMs and PMA-differentiated THP-1 cells stimulated by GM-CSF (**Supplementary Figure S3**). Collectively, these data strongly suggest that tiliroside promotes the macrophage balance towards an anti-inflammatory phenotype by inhibiting M1 polarization machinery, and this effect is dependent on LPS and IFNγ signaling.

### Tiliroside Regulates Macrophage Polarization *via* Blocking Glycolysis Pathway

**Figure 5:**
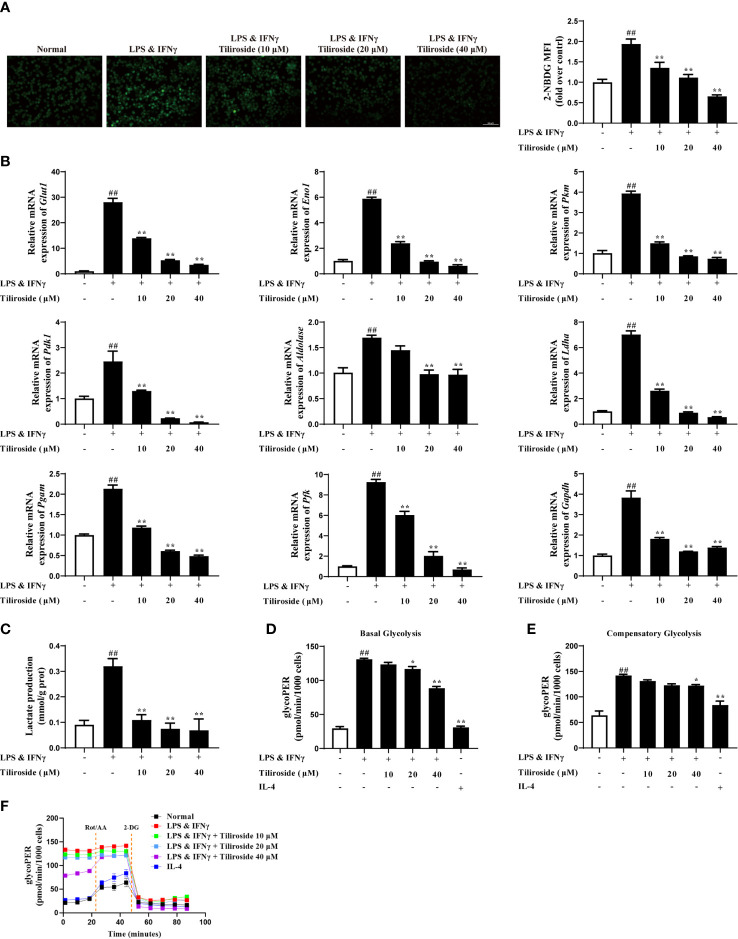
Tiliroside inhibits M1 polarization in a glycolysis-dependent manner. The BMDMs were treated with LPS (100 ng/ml) & IFNγ (20 ng/ml), tiliroside (10, 20, 40 μM) for 24 h. **(A)** The glucose uptake was analyzed by using an immunofluorescence assay (scale bar: 100 μm). **(B)** The mRNA expressions of *Glut1, Eno1, Pkm, Pdk1, Aldolase, Ldha, Pgam, Pfk*, and *Gapdh* were analyzed by using qPCR assay. **(C)** The production of lactate was analyzed by using the kits. **(D-F)** The basal glycolysis, compensatory glycolysis, ECAR, and mitoOCR/glycoPER basal ration were analyzed by using the seahorse. Data were expressed as means ± S.E.M of three independent assays. ^##^ *P* < 0.01 *vs.* Control group; ^∗^ *P* < 0.05 and ^∗∗^ *P* < 0.01 *vs.* LPS & IFNγ group.

The mechanism of tiliroside-mediated M1 macrophage regulation was investigated. Firstly, we tested whether tiliroside modulated the surface expressions of LPS sensors or IFNγ receptor. The results in **Supplementary Figure S4** showed that tiliroside did not influence the mRNA expression of *Cd14*, *Tlr4*, and *Ifngr*, suggesting that tiliroside may not have an effect on LPS/IFNγ sensing. As fatty acid oxidation (FAO) and glycolysis plays a key role in macrophage polarization (28), we then asked whether tiliroside halted M1 macrophage polarization through enhancing FAO or blocking the process of glycolysis. Herein, tiliroside (10, 20, and 40 μM) exerted little effect on the mRNA expressions of PPARγ targets genes including *Ap2* and *Cd36*, which has been demonstrated to play an important role in the process of FAO (**Supplementary Figure S5**). These data proposed the reasonable notion that the tiliroside-mediated macrophage polarization is less likely through FAO metabolism. Therefore, the effect of tiliroside on glycolysis was measured by monitoring the glucose uptake in LPS and IFNγ-stimulated BMDMs. As shown in **Figure 5A**, tiliroside (10, 20, and 40 μM) significantly decreased the uptake of 2-NBDG, a fluorescent deoxyglucose analog, which was widely used for monitoring cellular glucose uptake. Moreover, the mRNA expressions of glycolytic enzymes genes including glucose transporter 1 (*Glut1*), enolase 1 (*Eno1*), pyruvate kinase M (*Pkm*), pyruvate dehydrogenase kinase 1 (*Pdk1*), *Aldolase*, lactate dehydrogenase A (*Ldha*), phosphoglycerate mutase (*Pgam*), phosphofructokinase (*Pfk*), and glyceraldehyde-3-phosphate dehydrogenase (*Gapdh*) were markedly reduced by tiliroside in LPS- and IFNγ-stimulated BMDMs (**Figure 5B**). As a functional result, tiliroside inhibited the production of lactate in LPS and IFNγ stimulated BMDMs (**Figure 5C**). These inspiring findings prompted us to assess the metabolic characteristics of repolarized macrophages by extracellular flux analysis. As shown in **Figures 5D-F**, LPS and IFNγ clearly increased ECAR in BMDMs, while tiliroside treatment significantly decreased the basal glycolysis and compensatory glycolysis. Collectively, we propose the rational notion that tiliroside prevents classical M1 macrophage polarization *via* blocking glycolysis, and this inhibition effect was ascribed to disruption in the expressions of glycolytic enzymes.

### Tiliroside Dampens Glycolysis Through Enhancing HIF-1α Proteasomal Degradation

**Figure 6:**
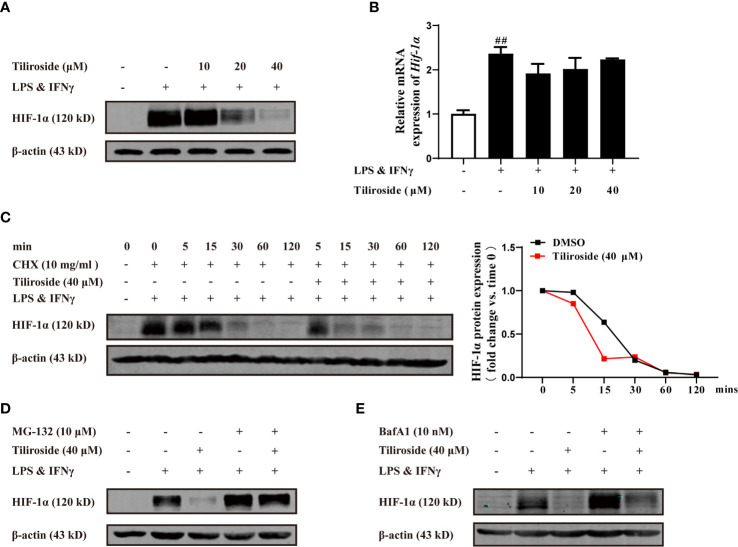
Tiliroside dampens glycolysis through enhancing HIF-1α proteasomal degradation. **(A, B)** The BMDMs were cultured with LPS (100 ng/ml) & IFNγ (20 ng/ml) and tiliroside (10, 20, 40 μM) for 24 h. The protein level of HIF-1α was analyzed by using western blotting assay **(A)**; the mRNA expressions of *Hif-1α* were analyzed by using qPCR assay **(B)**. **(C)** The BMDMs were treated with LPS (100 ng/ml) & IFNγ (20 ng/ml) for 24 h, and then treated with CHX (10 mg/ml) and tiliroside (40 μM) for the indicated periods. The protein level of HIF-1α was analyzed by using a western blotting assay. **(D, E)** The BMDMs were treated with LPS (100 ng/ml) & IFNγ (20 ng/ml) for 24 h, and then pretreated with MG132 (10 μM) or BafA1 (10 nM) for 2 h followed by incubation of tiliroside (40 μM). The protein level of HIF-1α was analyzed by using a western blotting assay. Data were expressed as means ± S.E.M of three independent assays. ^##^ *P* < 0.01 *vs.* Control group.

HIF-1α highly expresses in LPS and IFNγ stimulated macrophages (29) and serves as the major transcription factor regulating glycolytic enzyme expression (30). We thus hypothesized that tiliroside inhibited HIF-1α-mediated glycolysis to interrupt the M1 macrophage polarization and maintained the subsequent M1/M2 macrophage balance. As shown in **Figure 6A**, tiliroside (10, 20, and 40 μM) remarkably reduced the protein level of HIF-1α. To further determine the effect of tiliroside, we further measured the mRNA expression level of HIF-1α in LPS and IFNγ-activated BMDMs. Conversely, tiliroside exerted little effect on the mRNA expression of HIF-1α at the concentration of 10, 20, and 40 μM (**Figure 6B**). The further pulse-chase experiment indicated that tiliroside facilitated the turnover of HIF-1α protein in the presence of cycloheximide (CHX, 10 mg/ml), a broad-spectrum and nonspecific protein synthesis inhibitor (**Figure 6C**). Thus, it is reasonable to believe that the decrease of the HIF-1α protein level in tiliroside-treated BMDMs is due to increased degradation rather than decreased synthesis. Subsequently, proteasome inhibitor MG-132 and lysosome inhibitor BafA1 were employed to probe the potential pathways of tiliroside-mediated HIF-1α degradation. As shown in **Figure 6D**, MG132 treatment significantly inhibited the tiliroside-induced reduction of HIF-1α protein levels, while BafA1 treatment did not block the effect of tiliroside (**Figure 6E**). These findings collectively suggested that tiliroside regulated the expression of HIF-1α by the proteasomal degradation mechanism.

### Tiliroside-Mediated Inhibition of Colitis Is Macrophage Mediated

**Figure 7:**
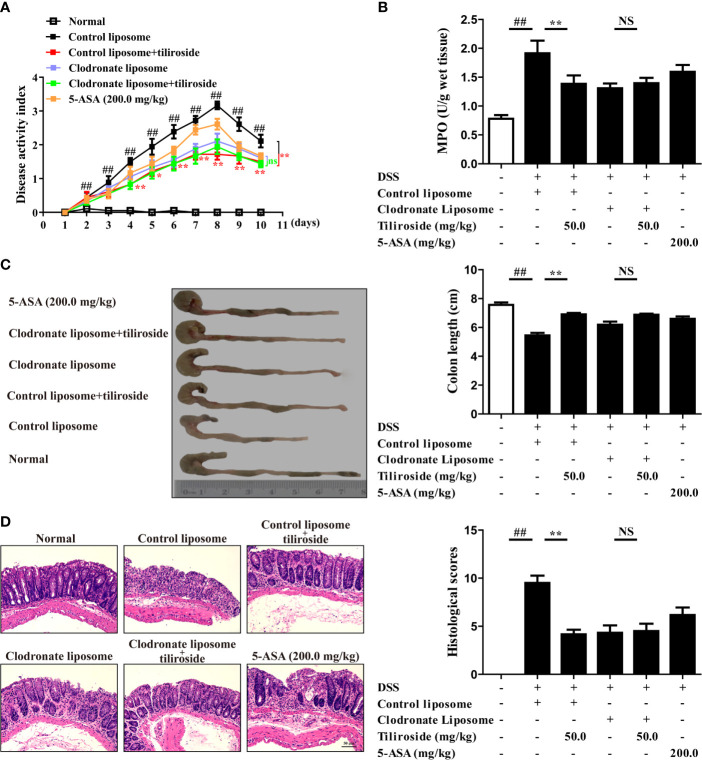
Tiliroside-mediated inhibition of colitis is macrophage dependent. C57BL/6 mice were subjected to DSS-induced colitis. Tiliroside (12.5, 25.0, and 50.0 mg/kg) and 5-ASA (200 mg/kg) were orally administrated daily for consecutive 10 days. The clodronate and control liposomes were intraperitoneally injected 12 h prior to the onset of DSS treatment and followed by injection every 48 h. At the end of the experiment, the mice were sacrificed, and the colons were collected. **(A)** The disease activity index (DAI) scores; **(B)** myeloperoxidase (MPO) activity in colons; **(C)** colon length; and **(D)** histopathological changes of colons were detected. Data were expressed as means ± S.E.M of six mice in each group. ^##^ *P* < 0.01 *vs.* Normal group; ns, not significant. ^∗∗^ *P* < 0.01 *vs.* Control liposome group.

Considering that tiliroside dampens M1 macrophage polarization through accelerating the proteasomal degradation of HIF-1α in BMDMs, we then determined whether macrophages were responsible for the tiliroside-mediated alleviation of colitis. We compared the colitis alleviation in the mice treated with tiliroside or tiliroside plus clodronate liposomes, which can significantly deplete macrophages *in vivo*. The DSS-induced colitis mice treated with control liposomes presented several colitis symptoms, evidenced by increased DAI scores, shorter colon length, increased MPO activity, serious pathological damage in colons, and tiliroside alleviated these symptoms. More importantly, the protective effect of tiliroside on colitis was not stronger in the presence of clodronate liposomes, suggesting that tiliroside inhibited colitis through a macrophage-dependent mechanism (**Figures 7A–D**).

## Discussion

**Figure 8:**
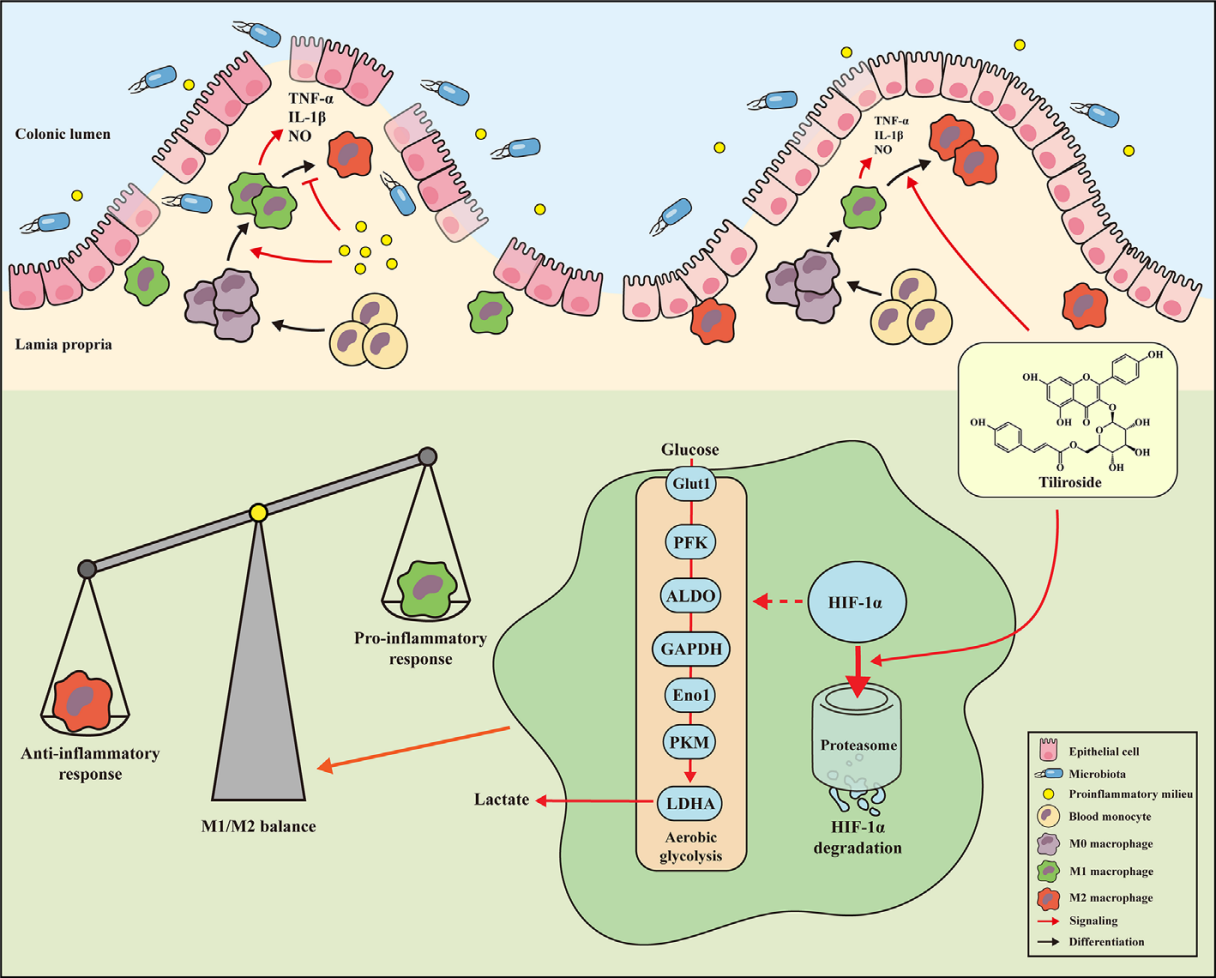
Mechanisms of tiliroside-mediated improvement of ulcerative colitis. Tiliroside dampens the HIF-1α/glycolysis pathway to restore the M1/M2 macrophage balance and attenuate the colitis.

The present study demonstrated for the first time that the small molecule tiliroside protected mice against DSS- and TNBS-induced acute colitis by regulating the M1/M2 macrophage balance towards an anti-inflammatory status. More importantly, tiliroside was found to directly promote the HIF-1α proteasomal degradation, leading to reduced expression of glycolytic enzymes, which in turn halted the polarization of M1 macrophage (**Figure 8**). Collectively, our findings demonstrate that HIF-1α-mediated glycolysis inhibition by tiliroside is responsible for remodeling M1/M2 phenotype balance and the prevention of UC. Our findings may help guide decisions regarding the use of tiliroside in patients with UC.

UC is an idiopathic and relapsing inflammatory disorder characterized by abundant inflammatory cells infiltrating into the mucosa lining of the colon and the rectum. Typical presenting symptoms include belly pain, abdominal cramping, fatigue, diarrhea, and bloody stool. At present, the chemical reagents, gene knockout, and transgenic mice-induced intestinal inflammation model are widely used in scientific research (31–33), among which DSS is the most frequently used chemical reagent to induce colitis. Firstly, DSS can induce intestinal inflammation with histopathologic and symptomatic features resembling human UC, including body weight loss, diarrhea, rectal bleeding, intestinal epithelial barrier damage, crypt architecture destruction, and infiltration of inflammatory cells into the mucosal tissues (34). Moreover, this model has the advantages of a high success rate and good repeatability (35). Additionally, there is an increasing number of researchers exploring the anti-colitis effect of related therapeutic agents by using DSS-induced colitis mice (34, 36). Therefore, we establish the DSS-induced colitis model in mice to investigate the effect of tiliroside. As expected, tiliroside (25.0 and 50.0 mg/kg) oral administration decreased the DAI scores in a dose-dependent manner, and the effect was even stronger than the commonly used anti-inflammatory drug 5-ASA (200.0 mg/kg). Additionally, tiliroside (25.0 and 50.0 mg/kg) prevented the shortening of colon length, reduced the activity of MPO in colons, allowed a relatively intact surface epithelium, minor loss of crypts, mild inflammatory cells in the mucosa, and less reduction of goblet cells compared to the DSS group. To further evaluate the therapeutic effect of tiliroside, another classical colitis model induced by TNBS in mice was used, and the results (**Supplementary Figure S1**) similarly showed that tiliroside dramatically improved survival rate, decreased MPO activity, lengthened the colons, and alleviated the histopathological damage in colons. The results suggest that tiliroside significantly attenuates the clinical symptoms of colitis.

Large bodies of studies have revealed that macrophages are one kind of the abundant leukocytes in the colon and the phenotype they adopt can markedly affect local mucosal innate and adaptive immune response. Traditionally, persistent activation of M1 macrophages contributes to excessive pro-inflammatory cytokines release, leading to the colonic homeostasis imbalance and barrier damage (37). In contrast, macrophage with an M2 phenotype can produce anti-inflammatory cytokines to ameliorate the progression of UC (38). More importantly, the proportion of M1 macrophages was significantly increased in inflamed colons of patients suffering from colitis, while the transfer of *in vitro-*generated M2 macrophages was shown to alleviate disease severity. The monoclonal antibodies targeting M1-associated cytokines TNF-α, including infliximab and adalimumab, have been approved to treat moderate to severe colitis in clinic (39, 40), while patients with the M2-related factor IL-10 receptor deficiency have enhanced susceptibility to severe colitis (41). Therefore, targeting macrophage polarization would be an effective strategy for controlling colitis, and our results suggest that tiliroside alleviates the development of colitis *via* regulating the balance between pro-inflammatory M1 and anti-inflammatory M2 macrophages (**Figures 2**, **3**). Previous seminal works (37, 42, 43) have provided a more rigorous analysis of the identity and functions of the lamina propria macrophage population (P1, P2, P3/P4). Mowat AM et al. have demonstrated that a monocyte to macrophage differentiation continuum exists in the intestinal lamina propria, a process that has been known as the monocyte “waterfall” (37). Biswas A, et al. have identified four different groups of macrophages based on the expression of Ly6c and major histocompatibility complex (MHC) II: P1 (Ly6c^hi^ MHCII^-^), P2 (Ly6c^in to hi^ MHCII^+^), and P3 + P4 (Ly6c^low^ MHCII^+^, P4 CX3CR1^+^). The P1 and P2 macrophages have pro-inflammatory characteristics, whereas P3 and, P4 macrophages have anti-inflammatory properties (43). Future study is necessary and warranted to further investigate the effect of tiliroside on lamina propria macrophage subpopulations. Nevertheless, our current study provides evidence to show that tiliroside inhibits colitis through a macrophage-dependent manner (**Figure 7**), and the effect was mainly achieved by regulating the balance of pro-inflammatory/anti-inflammatory macrophage *in vivo*. To further confirm our *in vivo* findings of tiliroside on macrophage polarization, BMDMs were treated with LPS & IFNγ or IL-4 in the presence or absence of tiliroside *in vitro* to directly examine macrophage polarization machinery. Our results showed that tiliroside significantly dampened the polarization of M1 macrophages and to a lesser extent increased the expression of M2 marker CD206 in mouse BMDMs and PMA-differentiated human THP-1 cells (**Figure 4**, **Supplementary Figure S2**) indicating that tiliroside functions to restore the balance between M1 and M2 macrophages.

Accumulating evidence suggests metabolic reprogramming can modulate macrophage activation. The metabolic signals not only provide energy but also regulate macrophage polarization. M1 macrophages are highly dependent on glycolytic metabolism, while M2 macrophages are mainly dependent on oxidative phosphorylation (44). Glycolysis is a metabolic pathway that converts glucose to pyruvate and lactic acid under the action of series enzymes in the cytoplasm. Knockdown of PDK1, a key regulator enzyme in glucose metabolism, dampened M1 but enhanced M2 activation of macrophages (45). Treatment with 2-DG, the inhibitor of glycolysis, attenuated the activation of M1 macrophages and the secretion of pro-inflammatory cytokines (12). In the present study, tiliroside significantly decreased the uptake of glucose and lactate production (**Figures 5A–C**), indicating the inhibitory effect on glycolysis (**Figures 5D–F**). Furthermore, tiliroside significantly downregulated the mRNA expressions of major enzymes thatparticipated in glycolysis (**Figure 5B**), indicating that tiliroside may affect a major signaling pathway upstream of glycolytic enzymes.

HIF-1 is a heterodimeric transcription factor that is composed two subunits: the HIF-1α and aryl hydrocarbon receptor nuclear translocator (Arnt, also named HIF-1β). Under normoxia condition, the HIF-1α is commonly hydroxylated at prolines 402 and 564 by prolyl-4-hydroxylase (PHDs), and the hydroxylated HIF-1α is then immediately ubiquitinated by E3 ubiquitin ligase degraded through the 26S proteasome pathway (46). In contrast, the PHDs are less active under hypoxic conditions, and the HIF-1α is more stable. This stabilization facilitates the HIF-1α to translocate into the nucleus and dimerize with the HIF-1β to form the complex, which then binds to the hypoxia-responsive elements (HREs) and activates transcription of hypoxia-responsive glycolytic gene, including PDK1 and Glut1 (47). More importantly, activation by LPS in combination with IFNγ appears to stabilize the HIF-1α subunits and induce metabolic reprogramming to glycolysis in M1 macrophages (48). Intriguingly, the present study demonstrated that tiliroside selectively dampened the protein stability of HIF-1α by regulating its ubiquitin-proteasomal degradation (**Figure 6**).

## Conclusion

The current study demonstrates that tiliroside may be an effective therapeutic approach to treat intestinal inflammation. This therapeutic effect is mediated by restoring the balance between M1 and M2 macrophages *in vivo*. In addition, our findings also provide new insight into the contribution of glycolysis to the tiliroside-mediated destruction of M1 macrophage polarization and suggest that targeting HIF-1α may be a potential therapeutic strategy for immune and inflammation-related diseases.

## Data Availability Statement

The original contributions presented in the study are included in the article/**Supplementary Material**. Further inquiries can be directed to the corresponding author.

## Ethics Statement

The animal study was reviewed and approved by Animal Ethics Committee of Jiangxi University of Traditional Chinese Medicine.

## Author Contributions

QL and JY designed the study and coordinated all experimental work. HZ, QL, YC, and LH carried out the experimental work. HZ, QL, CZha, and JY analyzed and interpreted the data. HZ, QL, CZho, and JY wrote the manuscript with valuable input from all other authors. All authors contributed to the article and approved the submitted version.

## Funding

This work was supported by grants from the National Natural Science Foundation of China (No. 31660328 and 81903885), and the Jiangxi Key Laboratory grant (No. 20202BCD42014), the Research Start-up Fund of Jiangxi University of Traditional Chinese Medicine (2018BSZR002), and the First-Class Discipline Development grant (JXSYLXK-ZHYI054).

## Conflict of Interest

The authors declare that the research was conducted in the absence of any commercial or financial relationships that could be construed as a potential conflict of interest.
