# Supplementary material for: Tiliroside Ameliorates Ulcerative Colitis by Restoring the M1/M2 Macrophage Balance via the HIF-1α/glycolysis Pathway
Source: Front Immunol. 2021 Mar 31;12:649463. doi: 10.3389/fimmu.2021.649463 (PMC8044352; doi:10.3389/fimmu.2021.649463)
Supplement: Supplementary file 1 [file DataSheet_1.pdf]

Supplementary Material

Tiliroside Ameliorates Ulcerative Colitis by Restoring M1/M2 Macrophage Balance *via* HIF-1α/glycolysis Pathway

Hongda Zhuang [1,†], Qi Lv [2,1†], Chao Zhong [1,3], Yaru Cui[1], Luling He[1], Cheng Zhang[3], Jun Yu[3,*]

[1]Translational Medicine Centre, Jiangxi University of Traditional Chinese Medicine, Nanchang, Jiangxi, 330002, China.

[2]Jiangsu Key Laboratory for Functional Substance of Chinese Medicine, Stake Key Laboratory Cultivation Base for TCM Quality and Efficacy, School of Pharmacy, Nanjing University of Chinese Medicine, Nanjing, 210023, China.

[3]Center for Metabolic Disease Research and Department of Physiology, Lewis Katz School of Medicine, Temple University, Philadelphia, PA 19140, USA

[†]These authors contributed equally to this work.

** Correspondence:*

Jun Yu, MD.

Center for Metabolic Disease Research and Department of Physiology,
Lewis Katz School of Medicine, Temple University,
3500 North Broad Street,
Philadelphia, PA 19140, USA
Email: jun.yu@temple.edu

Supplementary Tables 1. Primers used for RT-PCR

Primers	NCBI GeneID	Source		Sequence (5'→3')
β-actin	11461	mouse	Forward	CTACCTCATGAAGATCCTGACC
			Reverse	CACAGCTTCTCTTTGATGTCAC
Tnf-α	21926	mouse	Forward	CCCTCACACTCAGATCATCTTCT
			Reverse	GCTACGACGTGGGCTACAG
Il-1β	16176	mouse	Forward	GCAACTGTTCCTGAACTCAACT
			Reverse	ATCTTTTGGGGTCCGTCAACT
Inos	18126	mouse	Forward	GTTCTCAGCCCAACAATACAAGA
			Reverse	GTGGACGGGTCGATGTCAC
Arg1	11846	mouse	Forward	CTCCAAGCCAAAGTCCTTAGAG
			Reverse	AGGAGCTGTCATTAGGGACATC
Ym1	12655	mouse	Forward	CAGGTCTGGCAATTCTTCTGAA
			Reverse	GTCTTGCTCATGTGTGTAAGTGA
Cd206	17533	mouse	Forward	CTCTGTTCAGCTATTGGACGC
			Reverse	CGGAATTTCTGGGATTCAGCTTC
Glut1	20525	mouse	Forward	GCTTCCTGCTCATCAATCGT
			Reverse	CGACCCTCTTCTTTCATCTCC
Eno1	13806	mouse	Forward	TGCGTCCACTGGCATCTAC
			Reverse	CAGAGCAGGCGCAATAGTTTTA
Pkm	18746	mouse	Forward	AGACGGTGGACATAGTTGGC
			Reverse	TTTGCTGATGTTTTGCTTGC
Pdk1	228026	mouse	Forward	GGACTTCGGGTCAGTGAATGC
			Reverse	TCCTGAGAAGATTGTCGGGGA
Aldolase	11676	mouse	Forward	AAGGCTGCTCCATCAACACT
			Reverse	CACAGACAACACCGCACAC
Ldha	16828	mouse	Forward	CAAAGACTACTGTGTAACTGCGA
			Reverse	TGGACTGTACTTGACAATGTTGG
Pgam	18648	mouse	Forward	CAGGTAAAGATCTGGAGACGAT
			Reverse	CTTGCTGATGTTGCTGTAGAAG
Pfk	18642	mouse	Forward	GATGTTGTAGGTGCGGAGATTC
			Reverse	ACGGTATACATCGTGCATGAT
Gapdh	14433	mouse	Forward	AAATGGTGAAGGTCGGTGTG
			Reverse	TGAAGGGGTCGTTGATGG
Hif-1α	112405	mouse	Forward	ACCTTCATCGGAAACTCCAAAG
			Reverse	ACTGTTAGGCTCAGGTGAACT
Tlr4	21898	mouse	Forward	GCAAACGCTGTTCTGCTCAG
			Reverse	AGGCGTCTCCCTCTATTGTATT
Cd14	12475	mouse	Forward	CTCTGTCCTTAAAGCGGCTTAC
			Reverse	GTTGCGGAGGTTCAAGATGTT
Ifngr	15979	mouse	Forward	CTGGCAGGATGATTCTGCTGG
			Reverse	GCATACGACAGGGTTCAAGTTAT
Cd36	12491	mouse	Forward	ATGGGCTGTGATCGGAACTG
			Reverse	GTCTTCCCAATAAGCATGTCTCC
Ap2	11770	mouse	Forward	AAATCACCGCAGACGACAG
			Reverse	TCATAACACATTCCACCACCA
Cdh1	21872	mouse	Forward	CAGTTCCGAGGTCTACACCTT
			Reverse	TGAATCGGGAGTCTTCCGAAAA
Cldn7	53624	mouse	Forward	GGCCTGATAGCGAGCACTG
			Reverse	GTGACGCACTCCATCCAGA

Ocln	18260	mouse	Forward	TTGAAAGTCCACCTCCTTACAGA
			Reverse	CCGGATAAAAAGAGTACGCTGG
β-actin	60	human	Forward	CATGTACGTTGCTATCCAGGC
			Reverse	CTCCTTAATGTCACGCACGAT
Tnf-α	7124	human	Forward	CCTCTCTCTAATCAGCCCTCTG
			Reverse	GAGGACCTGGGAGTAGATGAG
IL-1β	3553	human	Forward	TTCGACACATGGGATAACGAGG
			Reverse	TTTTTGCTGTGAGTCCCGGAG
Inos	4843	human	Forward	GACTTTCCAAGACACACTTCAC
			Reverse	TTCGATAGCTTGAGGTAGAAGC
Arg1	383	human	Forward	GGACCTGCCCTTTGCTGACATC
			Reverse	TCTTCTTGACTTCTGCCACCTTGC
Cd206	4360	human	Forward	GACGTGGCTGTGGATAAATAAC
			Reverse	CAGAAGACGCATGTAAAGCTAC
Ccl18	6362	human	Forward	AGATCATTTACACAATGCTGGC
			Reverse	TAAAGTCACCAAAAGGCTTTCG

Supplementary Figure S1

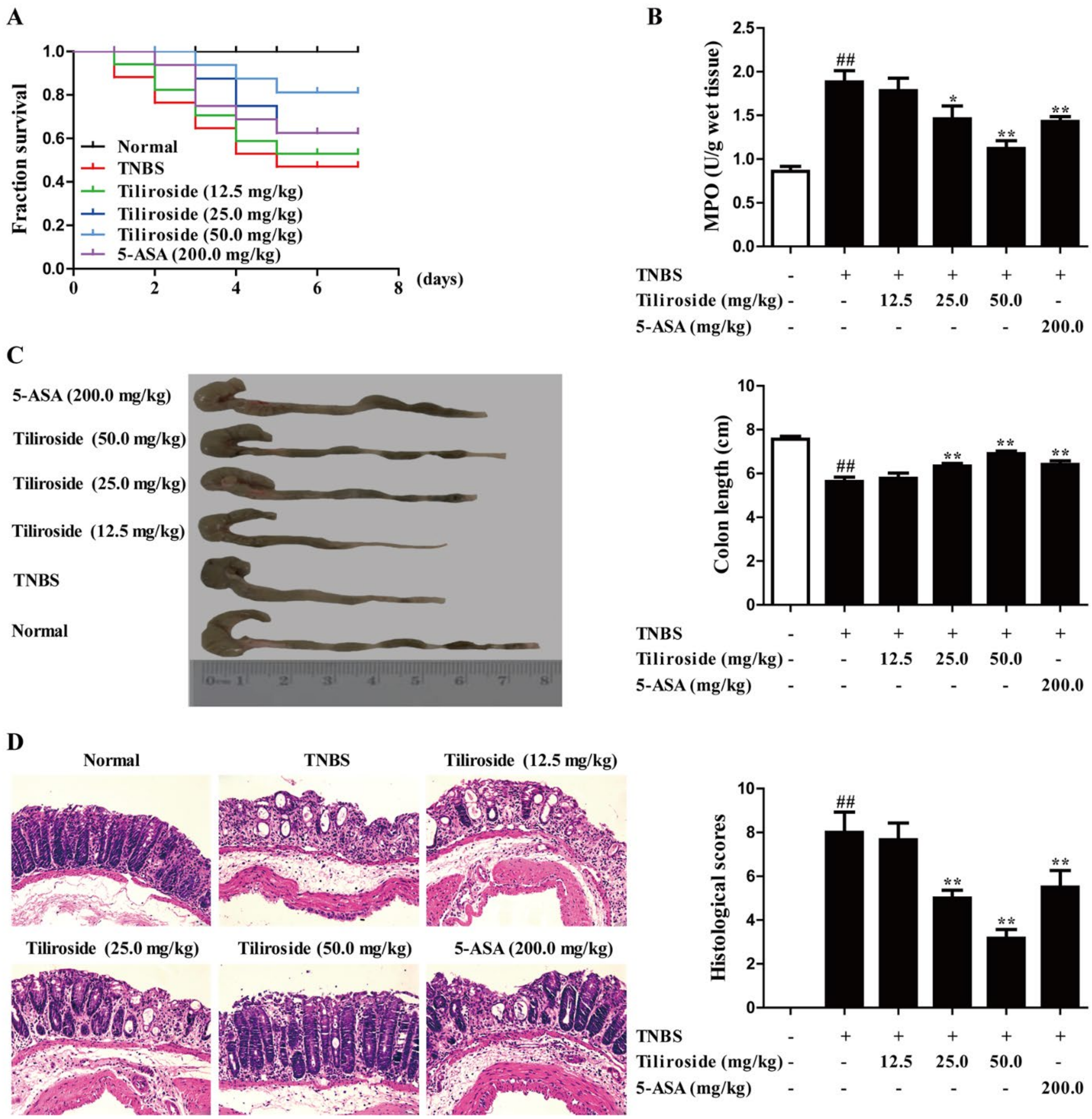

Supplementary Figure S1. Tiliroside alleviates TNBS-induced colitis in mice. The male BALB/c mice were challenged with TNBS, tiliroside (12.5, 25, 50 mg/kg) and 5-ASA (200 mg/kg) were orally administered daily from day 0 to day 7. (A) Fraction survival; (B) the MPO activity in colons; (C) the colon length; (D) the histological changes of colons were detected. Data were expressed as means ± S.E.M of fifteen mice in each group. $^{\#\#}P<0.01$ *vs.* Normal group. $^{*}P<0.05$ and $^{**}P<0.01$ *vs.* TNBS group.

Supplementary Figure S2

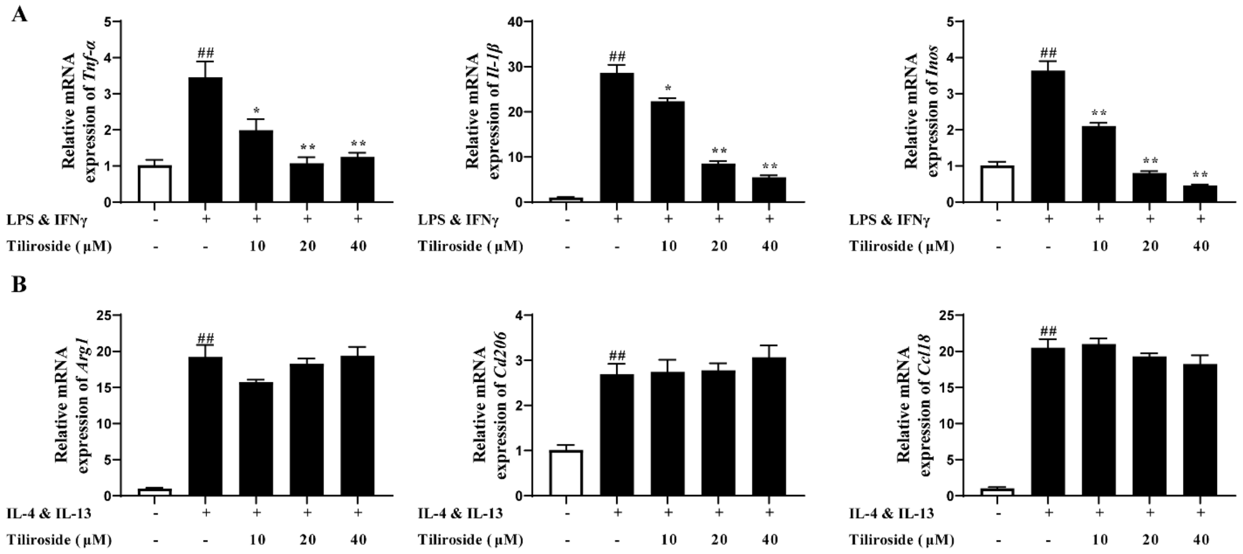

Supplementary Figure 2. Tiliroside dampens the polarization of M1 macrophage *in vitro*. (A, B) The PMA-differentiated THP-1 cells were treated with tiliroside (10, 20, 40 μM) in the presence or absence of LPS (100 ng/mL) and IFN-γ (20 ng/mL) or IL-4 (20 ng/mL), respectively. The mRNA expressions of M1-and M2-marker genes were detected by using the qPCR assay. Data were expressed as means ± S.E.M of three independent experiments *in vitro*. $^{\#}P < 0.05$ and $^{\#\#}P < 0.01$ vs. Normal group; $^{*}P < 0.05$ and $^{**}P < 0.01$ vs. LPS & IFN-γ group.

Supplementary Figure S3

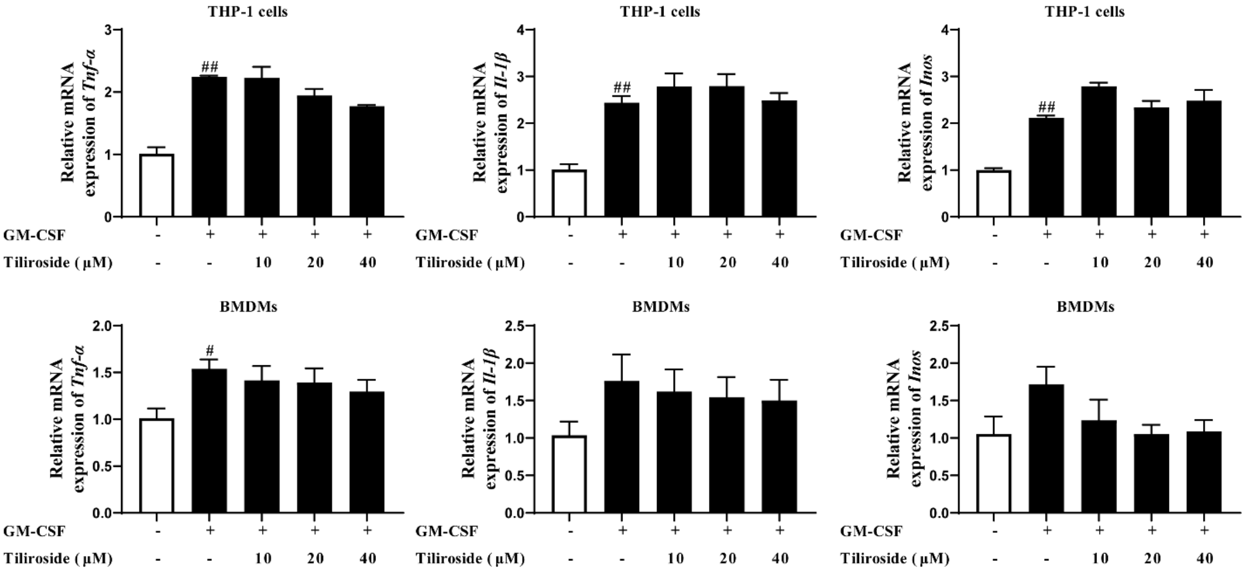

Supplementary Figure S3. The effect of tiliroside on the GM-CSF-mediated M1 macrophage polarization. The BMDMs and PMA-differentiated THP-1 cells were treated with the tiliroside (10, 20, 40 μM) for 24 h in the presence of GM-CSF (100 ng/mL). The mRNA expressions of *Tnf-α*, *Il-1β* and *Inos* were detected by using the qPCR assay. Data were expressed as means ± S.E.M of three independent experiments *in vitro*. $^{\#}P<0.05$ and $^{\#\#}P<0.01$ *vs.* Normal group.

Supplementary Figure S4

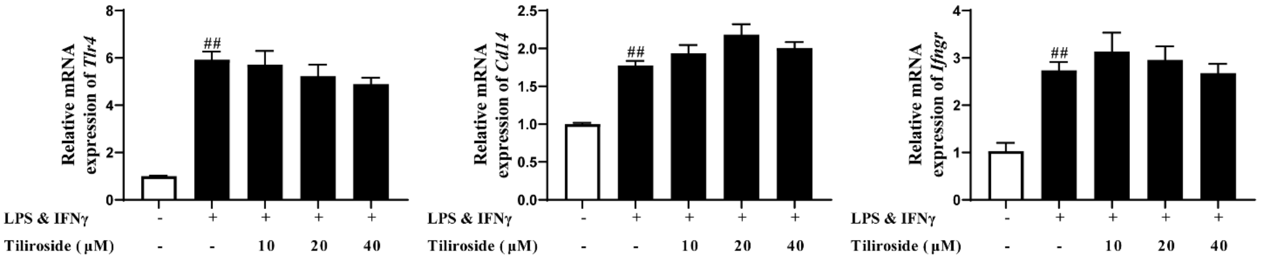

Supplementary Figure S4. Tiliroside exerts little influence on the mRNA expression of *Cd14*, *Tlr4* and *Ifngr*. The BMDMs were treated with tiliroside ((10, 20, 40 μM) in the presence or absence of LPS (100 ng/mL) and IFN- γ (20 ng/mL), the mRNA expression of *Cd14*, *Tlr4* and *Ifngr* were detected by using qPCR analysis. Data were expressed as means ± S.E.M of three independent experiments *in vitro*. $^{\#}P < 0.05$ and $^{\#\#}P < 0.01$ *vs*. Normal group; $^{*}P < 0.05$ and $^{**}P < 0.01$ *vs*. LPS & IFN-γ group.

Supplementary Figure S5

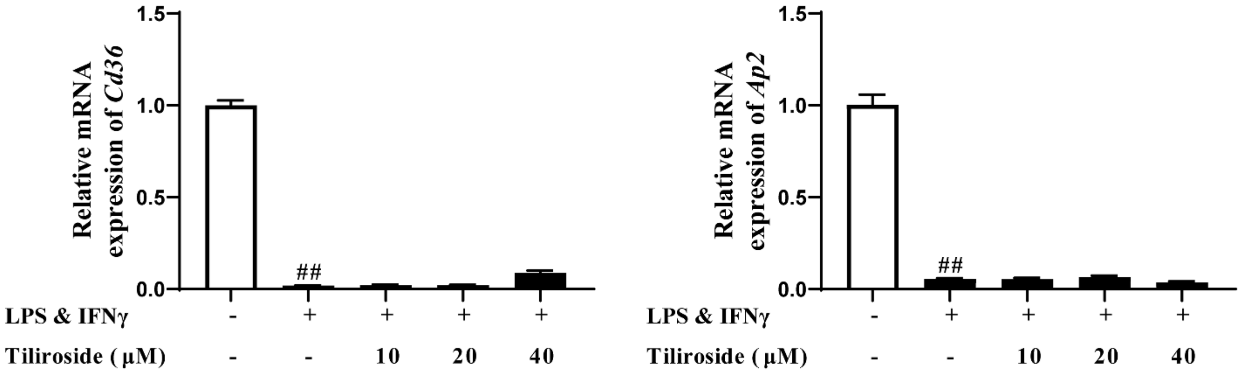

Supplementary Figure S5. Tiliroside exerts little effect on the PPARγ activation in macrophages. The BMDMs were treated with tiliroside (10, 20, 40 μM) in the presence or absence of LPS (100 ng/mL) and IFN-γ (20 ng/mL) for 24 h. The mRNA expressions of *Ap2* and *Cd36* was detected by using the qPCR assay. Data were expressed as means ± S.E.M of three independent experiments *in vitro*. $^{\#}P < 0.05$ and $^{\#\#}P < 0.01$ vs. Normal group; $^{*}P < 0.05$ and $^{**}P < 0.01$ vs. LPS & IFN-γ group.
